# Hygienic Behavior, Liquid-Foraging, and Trophallaxis in the Leaf-Cutting Ants, *Acromyrmex subterraneus* and Acromyrmex *octospinosus*

**DOI:** 10.1673/031.009.6301

**Published:** 2009-12-03

**Authors:** Freddie-Jeanne Richard, Christine Errard

**Affiliations:** ^1^ University of Poitiers, Laboratoire Ecologie, Evolution, Symbiose, UMR CNRS 6556, 40 avenue du Recteur Pineau, Poitiers, France; ^2^ Institut de Recherche sur la Biologie de l'Insecte, IRBI - UMR CNRS 6035, Faculté des Sciences et Techniques, Université François Rabelais, F-37200 Tours, France

**Keywords:** fungus-growing ants, grooming, fungus privation, foragers

## Abstract

Neotropical leaf-cutting ants (tribe Attini) live in obligate symbiosis with fungus they culture for food. To protect themselves and their fungus garden from pathogens, they minimize the entry of microorganisms through mechanical and chemical means. In this study, focusing on the species *Acromyrmex subterraneus* and *A. octospinosus,* (Hymeoptera: Formicidae). Self- and allo-grooming behavior were quantified and it was found that *A. octospinosus* workers spend less time in self-grooming than *A. subterraneus.* In the experimental absence of fungus in *A. subterraneus,* the times spent in these two behaviors are not affected; however workers spend significantly more time immobile. Hygienic and trophallaxis behaviors were examined as well as the possibility that workers exchange food, and the grooming behavior of foraging and non-foraging workers were compared. Behavioral observations revealed that large workers spent more time grooming than small workers, and more than 62% of replete foragers passed collected liquid food via trophallaxis to a nestmate. However, trophallaxis was rarely observed between non-forager workers. These results suggest that trophallaxis permits the exchange of alimentary liquid between colony members, but it is not important for spreading the colony odor signature.

## Introduction

Social insects, such as ants, honeybees, wasps and termites provide a highly favorable environment for the development of pathogenic bacteria, fungi, and other microbes. In order to prevent pathogen or disease transmission within the colony many social insects have developed various strategies including behavioral adaptations ( Cremer et al. 2007). Honey bees, for example, can detect and reduce the impact of bacteria, fungi or mites through hygienic behavior, which consists of removing infested brood from the wax cells (Spivak and Reuter 2001 and references therein). Fungus growing ants also perform hygienic behavior consisting of fungus raising and weeding, in which they remove parasitic fungi spores (*Escovopsis*) and other pathogens from the fungus garden (Currie and Stuart 2001). Social interactions, such as allo-grooming, also appear to decrease the susceptibility of both ant and termite colonies to disease (Hughes et al. 2002; Traniello et al. 2002).

Leaf-cutting ants (Formicidae: Myrmicinae, Attini) are restricted to the New World. They belong to a unique group of ants that are referred to as fungus-growers, because they cultivate symbiotic fungi for food. The genera *Atta* and *Acromyrmex,* which are the most phylogenetically derived fungus-growers, cut the leaves and flowers of a diverse collection of plant species. The size of *Acromyrmex* colonies can reach several hundreds of thousands of individuals, while colonies of their sister genus *Atta* can form colonies of to 5–10 million workers (Weber 1972; Hölldobler and Wilson 1990). Fungal cultivars serve as the primary food source for the ants, and they are carefully manured by the ants with foraged plant material, fruits, insect frass, or seeds. An additional energy source for leaf-cutting ant colonies comes from foragers drinking plant sap from the leaves that they cut (Littledyke and Cherrett 1976; Bass and Cherrett 1995). In a fungus-growing ants' colony, both the ants and fungus cultivar are exploited by pathogens (Currie et al. 1999a; Currie 2001a). The ants are known to minimize the entry of such micro-organisms by both mechanical means (licking, weeding) and chemical means via the production of antibiotics by the metapleural gland and symbiotic *Pseudonocardia* bacteria (Boyd and Martin 1975; Quinlan and Cherrett 1977; Currie et al. 1999b; Currie 2001b; Currie and Stuart 2001). The ant's infrabuccal pocket also produces antibiotics, which inhibit and kill spores of the parasite *Escovopsis* (Little et al. 2006). In addition, workers remove waste from fungus garden that minimizes potential pathogen development (Hart and Ratnieks 2001). Moreover, workers show aggressive behaviour directed toward nestmates contaminated with garbage (Hart and Ratnieks 2001). Indeed, absconding of the ants from their nest causes the fungus garden to be rapidly overwhelmed by alien microorganisms (Boyd and Martin 1975; Quinlan and Cherrett 1977; Currie et al. 1999a). The ants' foraging behaviors avoid harvesting of plant material with anti-fungal or other noxious compounds that might harm the cultivated fungus (Wiemer 1985; Howard and Wiemer 1986; Howard 1987, 1988; North et al. 1997).

Another challenge fungus-growing ants face for successful fungal cultivation is the contamination of garden by different strains of the symbiotic fungus, which is called “symbiont mixing” (Poulsen and Boomsma 2005). Different strains of the symbiotic fungus can result in incompatibility reactions, leading to a reduction in the yield of the fungus to the ants. This could occur through the accidental fusing of neighboring colonies or invasion by spores that are produced after the rare sporulation of the fungal symbiont in some colonies in the population. The domesticated fungi actively reject mycelial fragments of other strains of cultivar, and the strength of these reactions is in proportion to the overall genetic difference between these symbionts (Poulsen and Boomsma 2005). Fungus yield (and thus colony fitness) would thus be maximized if workers are able to distinguish between their own strain and alien strains of the fungal symbionts, which is a known behavior in leaf-cutting ants (Viana et al. 2001; Richard et al. 2007b), and this discrimination is correlated with between-colony differences in the chemical profile of the fungus (Richard et al. 2007a). This indicates that both genetic and environmental factors influence the fungus chemical profile. Discrimination between nestmates and non-nestmates appears to be based on a composite colony-specific “gestalt” profile common to all colony members (Crozier and Dix 1979; Fletcher and Michener 1987). This colony “gestalt” is maintained by the continuous exchange of recognition cues via trophallaxis and allo-grooming (Soroker et al. 1994), and it is affected, for example, by seasonal variation in diet (Nielsen et al. 1999; Silverman and Liang 2001; Richard et al. 2004) and nesting substrate (Heinze et al. 1996; Richard et al. 2007b). Grooming behaviour seems fundamental for passing of the colony odor within the colony (Meskali et al. 1995) via transfer of substances between workers, as, for example, in *A. subterraneus brunneus* (Camargo et al. 2006).

In spite of the workers' ability to recognize their own fungus, foragers are continuously exposed to many potential invasive alien fungal spores. Hygienic and allo-grooming behaviors appear to be very important as collective ant-parasite defenses in leaf-cutting ants. In this context, cleaning returning foragers seems more important than in non-fungus-growing ants. One aim of the present work, therefore, was to study the grooming behavior (self grooming and allo-grooming) in leaf-cutting ants.

We hypothesized that foragers returning from the external environment into the colony should spend more time engaging in grooming behavior than do non-foraging workers. To test this, hygienic behaviors were quantified and compared for two leaf-cutting ant species, *Acromyrmex subterraneus* and *A. octospinosus.* Due to the high polymorphism between workers and previous studies on the division of labor (Wilson 1980a, 1980b), workers were subdivided into two size categories (small and large). Non-foragers' grooming behaviors were compared with the fungus garden present and without the fungus garden for *A. subterraneus,* which was the species with the higher frequency of grooming behaviors. In order to examine the role of fungus in workers hygienic behavior the behavior of workers maintained in the nest was observed with and without fungus. Grooming and social interactions of replete and non-replete foragers that had fed on liquid foods were then compared. In many social insects this liquid food is stored in the foragers'crops, and it is regurgitated to nestmates once workers return to the nest. Regurgitation of plant sap and fruit juice would occur via trophallaxis (passing liquid from one ant to another, mouth-to-mouth), however this behavior had never been quantified in leaf-cutter ants, probably due to the difficulty of observing behaviors in the fungus garden.

## Materials and Methods

One monogynous (*i.e.,* a single mother queen per colony) laboratory colony was used of each of two species: *Acromyrmex subterraneus subterraneus* (collected in 1998 in Vicosa, Matto Grosso, Brazil) and *A. octospinosus* (collected in 2002 in Gamboa, Panama). The colonies had been transported to France, and each was kept in a dark plastic box that was linked by a tube to an external “foraging arena” containing food (fresh leaves of roses, brambles, and privet; orange juice; pieces of orange) and water, in a room with a temperature of 25° ± 2° C, 75% RH, and a photoperiod of 12 h light (between 18:00 to 06:00) vs. 12 h dark (06:00 to 18:00). Except during the experiments, the main nest box was completely covered by opaque cardboard. All colonies and sub-colonies were humidified every day using cotton saturated with water on the top of the nest and were supplied with the same diet.

## Baseline grooming behaviors

The experiments were performed on one queenless subcolony fragment for each species in June and July 2002. A ∼50 cm3 fragment was removed from the fungus garden of each colony and taken apart to collect light brown (i.e., young but fully mature) workers from inside the fungus for marking. The number of workers per fragment was 680 (19% larger majors, 29% medias, 52% smaller minors). The fungus and workers were then installed in a plastic box (“main nest box”; dimensions 18 × 12 × 5.8 cm) that was linked by a tube to an external “foraging arena” (28 × 27.5 × 9 cm) containing food and water. For future behavioral observations, we marked a total of 90 workers (60 larger workers, 30 smaller workers, henceforth referred to as LW and SW, respectively) in *A. subterraneus* and 50 workers (30 LW, 20 SW) in *A. octospinosus,* with an individual color mark (UniPaint PX-20, Mitsubishi Pencil Co. Ltd., www.uniball.com) on the thorax and/or on the abdomen. The colonies were subsequently left undisturbed for one week, apart from daily feeding and frequent (3–4 times per day) visual inspection of the foraging arena.

One week after marking, several marked workers were observed in the foraging arena, signifying that they had matured to an age for foraging. Thereafter the nest entrance was closed 30 minutes before nest observations in order to ensure that no foragers returned to the nest during observations. The nest entrance was left open overnight. Focal observations were made of marked workers on the surface of the fungus garden every day between 9am – 12pm and 2pm – 4pm. A focal worker was always observed for a continuous 30 min. In the rare event (< 5% of observation bouts) that the focal worker disappeared from view for more than 30 sec, a new 30 min observation period was started on another focal worker and the data from the aborted bout was discarded. In total, 60 workers (40 LW, 20 SW) from *A. subterraneus* and 30 workers (20 LW, 10 SW) from *A. octospinosus,* were observed excluding aborted bouts. A Psion II Organizer was used to score the frequency and duration (in seconds) of the following behaviors: 1. “giving or receiving trophallaxis” (the focal worker passed visible liquid to a sister worker through mouth-to-mouth contact); 2. “social behaviour” (allo-grooming, licking or antennating over any body part of a sister worker); 3. “self-grooming” (mouthparts were moved over her own antennae or legs, or rubbed two legs against each other); 4. “interacting with fungus” (licking, picking up, or re-arranging the symbiotic fungus); 5. “immobility” (the focal worker was resting immobile); and 6. “other activities” (any other behaviors, e.g., walking, interacting with the brood).

## Experiment 1: Grooming behaviors with and without fungus

**Figure 1.**
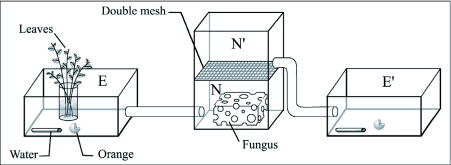
Experimental set-up for the fungus-less worker observations.N : nest area with the fungus, workers and queen and E: foraging area of workers belonging to the nest N; N': nest area with only workers and E': the foraging area of workers belonging to the nest N'.

These observations were observed only on *A. subterraneus.* The fungus garden was divided into two equal fragments of approximately a half litre. One fragment, including the queen, was installed in box N (Figure 1). In the second fragment, all workers were taken from the fungus garden and placed in box N' (Figure 1). These “fungus-less workers” were separated from the main nest (which contained the fungus garden and the majority of the workers) by a double wire mesh to prevent social grooming and exchange between the two fragments but not fungus odor diffusion. 10 SW and 22 LW (hereafter “fungus-less workers”) were observed in box N'.

## Experiment 2: Grooming behaviors of foragers that collect liquid foods

This experiment was done on *A. subterraneus* and *A. octospinosus* colonies in February and March 2004. For each of these colonies, we marked all “foragers” (exclusively LW, which were observed at any time in the foraging arena over a period of 2 days) with an individual color mark on the thorax (see Experiment 1). After marking, the colonies were left undisturbed for one week, but they did not receive any food during the last two days of this week. Subsequently, a single droplet (volume ca. 1ml) of orange juice was deposited in the foraging arena, which was otherwise empty of food. After the first marked forager to entered the foraging arena it was observed continuously. It could either drink from the droplet and subsequently return to the main nest (“replete forager”) or return to the main nest without drinking (“empty forager”). Immediately after it returned into the main nest, the nest entrance was blocked and it was observed for a continuous 10 min, following the same protocol as in Experiment 1. The nest entrance was subsequently reopened, the remains of the previous droplet were removed, and a fresh droplet was placed in the same location. The procedure described above was then repeated. In total, 35 replete foragers and 25 empty foragers were observed (spread out over 6 days) in two different times for the two species *A. subterraneus* and *A. octospinosus.*

## Statistics

All statistical tests were done with JMP software. All results are provided as mean ± SEM, and p-values are two-tailed unless otherwise stated. Effects of castes (i.e. minors or majors) and groups (i.e. species) were assessed with parametric ANOVAs after an Arcsine transformation of the data, and followed by Tukey's HSD post-hoc analyses for multiple comparisons (group, caste, and group x caste). The arcsine transform was used to fit data expressed as percents to the requirements of parametric tests. Finally, statistical assessment of the effect of species in ants' trophallaxies duration inside the nest, and workers' castes in ants raised without fungus were done using non-parametric Mann-Whitney *U*-tests on ranks since these data did not follow a normal distribution.

## Results

### Grooming and social interactions according to species and workers' size

**Figure 2.**
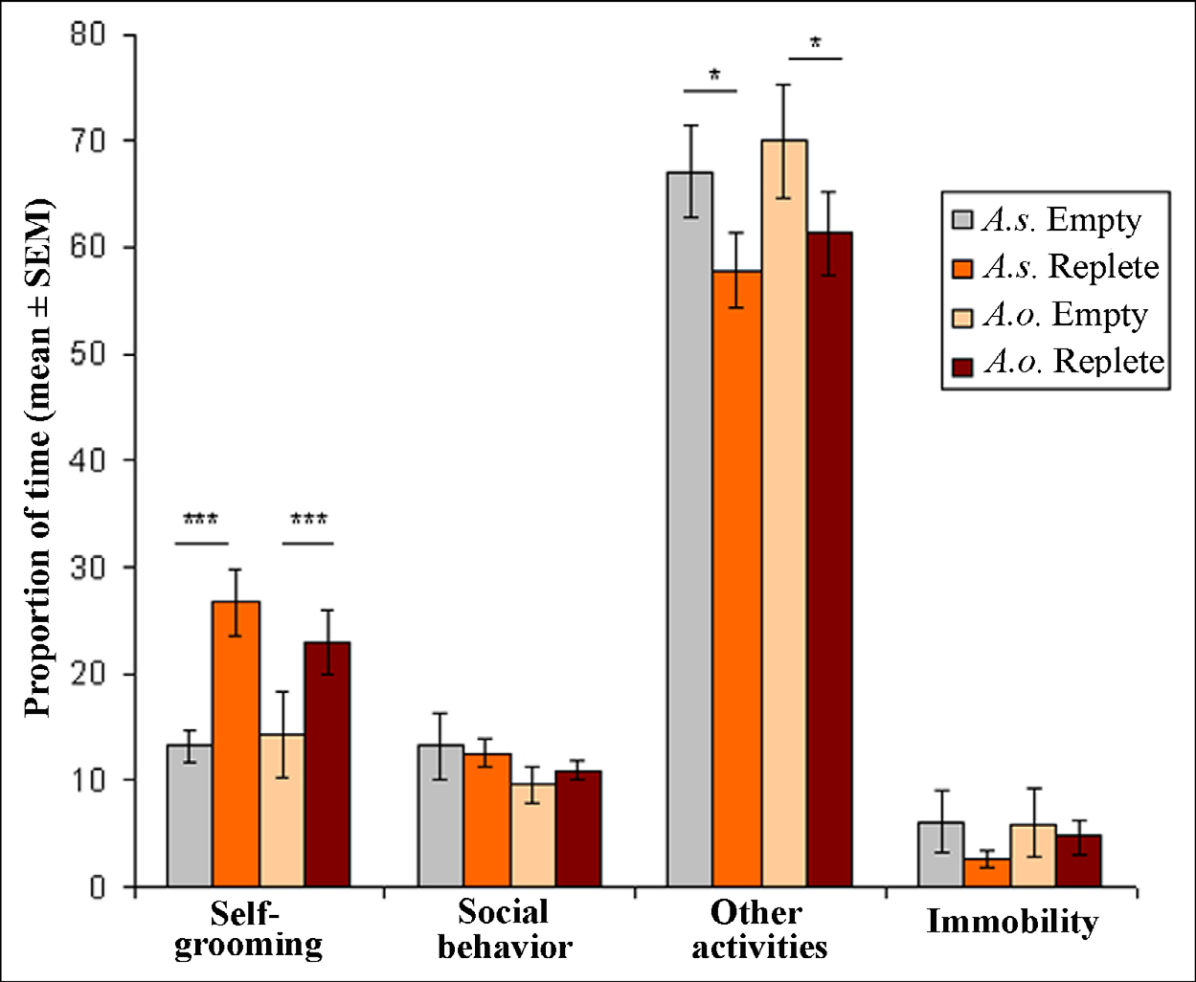
Relative proportion of each behavior (in %) of *Acromyrmex subterraneus* ants and *A. octospinosus* returning foragers in the nest. Returning foragers are separated in two groups, “replete” or “empty”. Data are presented as Mean ± SEM of behavior in percentage. Bars above columns represent groups being compared; astericks indicate level of significance, * = p<0.05; *** = p<0.01; *** = p<0.001.

In both species, large workers spent significantly more time expressing “self-grooming” than small workers (F (2,89) = 16.56, p <0.001, Tables 1 and 2). The total time the colony spent in self-grooming was significantly higher in *A. subterraneus* than in *A. octospinosus* (F (2,89) = 22.63, p <0.001) with no interaction effect between size and species (F (2,89) = 0.06, p = 0.79). Likewise, large workers spent significantly more time expressing “social behaviour” (i.e. antennal contact and allo-grooming) than small workers (F (2,89) = 3.77, p = 0.05) with no significant difference between both species (F(2,89)=0.88, p=0.35). Small workers spent significantly more time in “interacting with fungus” than large workers (SW: 22.19 ± 3.8 sec; LW: 8.5 ± 2.5 sec; F (2,89) = 6.55, p = 0.01). The time spent in contact with the fungus appeared equivalent between the two species (F (2,89) = 0.76; p = 0.38) with no interaction between species and workers' size (F (2,89) = 2.55, p = 0.11). In both species, small workers spent less time in “other activities” than large workers (F (2,89) = 4.6, p = 0.03) with no difference between species (F (2,89) = 0.97; p = 0.32) and no interaction effect (F (2,89) = 0.43, p = 0.41). Likewise, the amount of time “being immobile” did not differ between small workers and large workers (F(2,89)=0.32, p=0.57). The total time all workers spent in immobility was significantly lower in *A. subterraneus* than in *A. octospinosus* (F (2,89) = 8.67, p = 0.004 respectively, Figure 2) with no interaction effect (F (2,89) = 0.44, p = 0.50).

Trophallaxis was infrequent when the nest entrance was closed, occurring only 19 times during the 60-h of observation in *A. s. subterraneus,* and 4 times during the 30-h of observation in *A. octospinosus.* The mean duration of trophallaxis was 15 ± 7 sec (n = 19) in *A. s. subterraneus* and 7 ± 3 sec (n = 4) in *A. octospinosus* and did not differ significantly between both species (U = 21, p = 0.16).

### Grooming and interactions among *A. subterraneus* nest workers with or without fungus

The observation of *A. subterraneus* workers in the nest without fungus revealed a significant difference between the two castes: large workers engaged in more “social behaviors” than small workers, although this difference was marginally significant (U = 56; p < 0.08). There was no significant difference between LW and SW for any of the other scored behaviors (Us >101; p >0.7). LW without fungus garden spent significantly less time on self-grooming and more time being immobile compared to LW inside the fungus garden (F (2,91) = 4.02, p = 0.009 and F (2,91) = 6.02, p = 0.0007, respectively; Tukey's HSD post-hoc tests p <0.05), but there was no differences in social behaviors (F (2,91) = 1.2; p = 0.29). SW without fungus garden spent less time in “other activities”, and more time being immobile compared to SW inside the fungus garden (F (2,91) = 6.8, p=0.003 and F (2,91) = 6.02, p = 0.0007 respectively, Tukey HSD post-hoc tests p's <0.05).

At the colony level (i.e. pooling all workers with both sizes), the absence of fungus altered the time spent in self-grooming (F (2,91) = 4.22, p = 0.04) but not in social grooming (F (2,91) = 0.9, p = 0.34). In the absence of fungus, workers spent significantly more time immobile (F (2,91) =18.2, p <0.001) and also decreased the expression of all other activities (F (2,91) = 19.1, p <0.001).

**Table 1.**
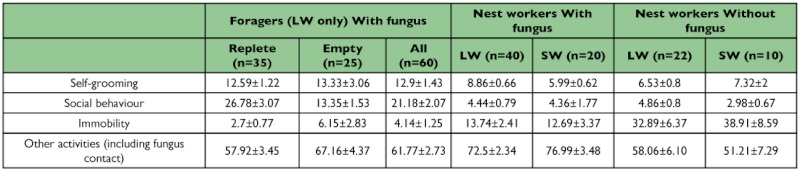
Behaviors displayed by A. *subterraneus* ants with and without fungus. Data are presented as Mean ± SEM of behavior duration in seconds.

**Table 2.**
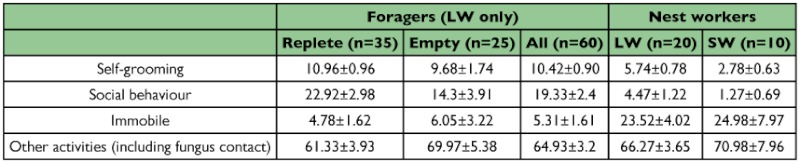
Behaviors displayed by A. *octospinosus* ants with fungus. Data are presented as Mean ± SEM of behavior duration in seconds.

### Grooming and interactions between returning foragers and nest workers

Leaf-cutting ant foragers regurgitate the liquid food collected while foraging to nest-mates through trophallaxis. Only replete foragers were observed performing such trophallaxis after their return into the nest. The percentage of replete foragers that engaged in trophallaxis reached 63% for *A. subterraneus,* and 67% for *A. octospinosus.* The mean duration of time spent in trophallaxis by returning replete foragers was 62 sec ± 34 (n = 22) in *A. s. subterraneus* and 66 sec ± 25 (n = 24) in *A. octospinosus* with no significant difference (F(1,45)=0.05, p = 0.82).

Replete foragers spent significantly more time in “social behaviors” than empty foragers (F(2,119)=76.4, p<0.001, Figure 2), with no significant species' or interaction effect (F(2,119)=0.04, p=0.84 and F(2,119)=0.02, p=0.87 respectively). Moreover, no significant difference was found between both types of foragers or both species concerning the time spent in self grooming, nor was there any species effect (all F(2,119)>5, all p >0.05).

The scored behavior of the marked foragers (all large workers) was compared between the earlier situations A (“returning foragers”, i.e. immediately after returning from a foraging tour trip into the main nest, irrespective of whether they were replete or empty) and the previous situation B (“confined LW”, i.e. non-foragers). Before analyses, the durations spent on each scored activity were normalized. Large workers engaged significantly more in “social behavior” and self grooming when “returning foragers” than when confined (F (2,182) = 80.6, p <0.001, Tables 1 and 2). Moreover, there was no significant difference between both species for these variables (F (2,182) = 0.02, p = 0.88, Table 1 and 2). Likewise, large workers spent more time in self-grooming as “returning foragers” than “non-foragers” (F (2,182) = 351.5, p <0.001). *A. subterraneus* workers spent significantly more time in self-grooming than *A. octospinosus* workers (F (2,182) = 21.5, p <0.001). Moreover, an interaction was observed between species and status: *A. subterraneus* foragers spending more time in self-grooming than *A. octospinosus,* and foragers of both species spending more time than non-foragers of both species in self-grooming (F (2,182) = 20.9, p <0.001). Finally, “Returning foragers” spent significantly less time being immobile than “non-foragers” (F (2,182) = 149.5, p <0.001). Moreover, *A. octospinosus* workers spent significantly more time being immobile than *A. subterraneus* workers (F (2,182) = 8.37, p <0.01). A significant interaction occurred between species and worker's status (F (2.182) = 8.32, p <0.001), as *A. subterraneus* non-foragers spent more time immobile than *A. octospinosus* non-foragers, and foragers of both species spent less time than non-foragers of both species being immobile.

## Discussion

### Grooming and social interactions

The comparison of worker behaviors inside and outside the nest showed significant differences in the length of time spent engaging in grooming behavior between the different worker castes. Inside the nest and in the presence of a fungus garden, large workers spent more time on grooming (self- and allo-grooming) than small workers for both *A. subterraneus* and *A. octospinosus.* The foragers displayed more self-grooming than the non-foragers.

Caste and division of labor in leaf-cutting ants are, in part, size-dependent (Wilson 1980a, 1980b; Andrade et al. 2002). Minor workers are usually involved in nest activities, especially fungus gardening. The fungus gardening includes substrate preparation by licking, chewing, crimping, and simultaneously, sterilizing it by applying antibiotics (Boyd and Martin, 1975; Quinlan and Cherrett, 1977; Currie et al., 1999b; Currie, 2001b; Currie and Stuart, 2001; Little et al. 2006). This sanitation behavior may help eliminate environmental chemicals, microorganisms, and/or help update the colony odor. By comparison, large workers spent less time on maintaining the fungus garden (i.e., “direct fungus contact”) than small workers. The finding that foragers displayed more self-grooming than non-foragers emphasizes the role of hygienic behavior and grooming in preventing the introduction of pathogens.

Being deprived of direct contact with the fungus garden increased the immobility time of both castes. The time spent in the other behaviors was not significantly affected by the presence or absence of the fungus garden, except that large workers spent more time on self-grooming in the presence of the fungus. In this experiment, volatile chemical fungal compounds could circulate easily by diffusion, so the differences are due to direct contact with the fungus rather than perception of the presence of the fungus via volatile odors.

### Trophallaxis

Workers of leaf-cutting ants were observed engaging in trophallaxis behavior. Specifically, foragers that brought back liquid food engaged more frequently in trophallaxis with nest workers than did returning “empty” foragers. Returning liquid foragers spent more time self-grooming, more time engaged in social behaviors, and less time immobile than did non-foragers. Trophallaxis between ants, both of which were non-foragers, did occur but was rare.

63% and 67% of replete foragers engaged in trophallaxis respectively in *A. subterraneus* and *A. octospinosus.* A concurrent study also found a very high percentage of trophallaxis (38%) in *A. subterraneus* (Moreira et al. 2006), especially compared to other species such as 22% in *Acromyrmex subterraneus subterraneus* (Kukuk and Crozier 1990) and 33% in *Cataglyphis iberica* (Dahbi et al. 1999). Moreover, in the present experiments, the mean time of the trophallaxis was about 65 seconds for both species, compared to 136 seconds reported in Moriera et al (2006)

The low frequency of trophallaxis between non-foragers has an interesting relationship to the presence of the fungus garden. The fungus, which is an essential source of food for the ants (Quinlan and Cherrett 1977; Hölldobler and Wilson 1990), secretes liquids (“fungus droplets”) that are rich in carbohydrates (≊ 14 µg/µl) and lipids (≊ 0.7 µg/µl) in *A. subterraneus* colonies (Richard, unpublished data). So the ants could feed on the fungus droplet even if they have not foraged on the fruit juice that was also available. Non-forager workers sometimes regurgitated a droplet onto the surface of the fungus (personal observation), after which another worker would be able to drink it. The non-forager workers rarely, however, engaged in trophallaxis. Thus, trophallaxis seems to take place with foraged liquids rather than with liquids generated endogenously within the nest. Considering that this study was conducted under laboratory conditions, a field study would be necessary to consolidate the importance of trophallaxis in *Acromyrmex* leaf-cutting ants.

It has been hypothesized that, in the social Hymenoptera, trophallaxis served as appeasement behavior during social tension and subsequently evolved as a major means for the distribution of liquid food (see Liebig et al. 1997). The ability to perform trophallaxis could be similarly important to the leaf-cutter ants, given that they also feed on plant sap and nectar from floral and extra-floral nectaries (Murakami and Higashi 1997). However, the mechanism of regurgitation present in leaf-cutting ants could have different functions. Our results suggest that trophallaxis can have a major role in the distribution of liquid food.

This study clearly indicates that workers' time spent in hygienic behavior and grooming, especially foragers, is a behavioral response that can prevent pathogens' entrance into the colony as well as pathogen development, but these behaviors also could be a way to maintain the homogeneity of the colony odor. However, trophallaxis seems to occur mainly in the context of liquid food distribution.

## Abbreviations

**LW**: large workers,
**SW**: small workers
